# Synthesis and characterization of fluorescence poly(amidoamine) dendrimer-based pigments

**DOI:** 10.1038/s41598-022-19712-5

**Published:** 2022-09-07

**Authors:** Marzieh Golshan, Behnam Gheitarani, Mehdi Salami-Kalajahi, Mahdi Salami Hosseini

**Affiliations:** 1 Faculty of Polymer Engineering, Sahand University of Technology, P.O. Box 51335-1996, Tabriz, Iran; 2 Institute of Polymeric Materials, Sahand University of Technology, P.O. Box 51335-1996, Tabriz, Iran

**Keywords:** Chemistry, Photochemistry, Polymer chemistry

## Abstract

In this work, we looked at how to make fluorescence hybrid poly(amidoamine) dendrimer (PAMAM) dendrimers using calcozine red 6G and coumarin end groups. After synthesis of ethylenediamine (EDA)-cored 4th generation PAMAM dendrimer (G4.0), surface functional groups is reacted with calcozine red 6G (Rh6G) and 7-methacryloyloxy-4-methylcoumarin. Fourier transform infrared spectroscopy, proton nuclear magnetic resonance (^1^H NMR), and X-ray diffraction are used to characterize the structure of synthesized fluorescent hybrid dendrimers. Optical properties are demonstrated using a fluorescence spectrophotometer, and UV–Vis–NIR reflectance spectra. According to UV–Vis–NIR reflectance spectra, hybrid dendrimers were transparent in the NIR range. Moreover, quantum yield (*Φs*) of hybrid dendrimers was calculated in dimethylformamide (DMF), ethanol, dimethyl sulfoxide (DMSO), and distilled water (H_2_O). Dendrimers in which Rh6G was utilized to modification showed the maximum quantum yield in ethanol due to great interaction of structure with ethanol and the arrangement of ring-opened amide shape of calcozine red 6G.

## Introduction

Visualizing the morphological details of cells has made bioimaging a powerful tool over past two decades^1,2^. Fluorescent probes are used to label specific chemical structures due to their selectivity and high sensitivity in bioimaging^3–5^. Many organic fluorescent dyes, including coumarin, rhodamine, and perylene have been developed for imaging and fluorescence detection; however, poor aqueous solubility of these organic fluorophores has limited their application in biological imaging^6–9^.

Coumarin and its derivatives, which are inartificial and artificial, are widely found in nature. They are used in the different areas such as medicine, biology, physics, and chemistry and the different applications such as nonlinear optical chromophores, laser dyes, fluorescent probes, fluorescent whiteners, and solar energy collectors due to their remarkable luminescence and optical features^10–18^. In recent years, the use of coumarins in polymer science has increased considerably and continues to develop due to their interesting properties, which are important for many fields such as organic/inorganic hybrid materials, electro-optical materials, biochemicals, liquid crystalline materials, and energy transferring/light harvesting materials^19–22^. In addition to their importance as the brightest fluorescent dyes, coumarins are one of the most widespread fluorescent groups. The most important fluorescence colors of coumarin have been reported to be yellow or green, whereas they have absorption and emission in the most parts of the visible spectrum. Another coumarin derivative is 4-methyl-7-hydroxycoumarin. This substance can create strong green–blue fluorescence through hydrogen bonds in alcohol and water solvents. A solution of 4-methyl-7-hydroxycoumarin in water produces a strong fluorescence at 450 nm^22^.

Calcozine red 6 dye is one of the most widely used organic dyes and has been extensively used for chemosensing^23,24^ and bioimaging^25^, owing to the high molar extinction coefficient, excellent photostability relative to other organic dyes, high fluorescence quantum yield, as well as its good water solubility.

Dendrimers are regular, monodisperse, and branched macromolecules with a single core molecule, layers of branched monomers, and numerous terminal groups^26,27^. Among dendrimers, the fluorescence emission of PAMAM dendrimers is the result of the presence of amidic acid, imine, and tertiary ammonium groups. However, fluorescent core dendrimers are a new class of polymeric materials with diverse applications. Prasad et al.^28^ studied the solvent-induced aggregation and its impact on the intrinsic emission properties of amine-, hydroxyl-, and carboxyl-terminated (PAMAM) dendrimers in C_3_H_8_O_3_, (CH_2_OH)_2_, CH_3_OH, C_2_H_4_(NH_2_)_2_, and H_2_O. The inherent emission properties of PAMAM dendrimers were changed dramatically in different solvents. When activated at 370 nm, an amine-terminated PAMAM dendrimer generated a strong emission at 470 nm in glycerol, ethylene glycol, and glycerol–water mixtures. Dodangeh et al.^29^ synthesized four new dispersible dyes made up of PAMAM dendrimer and thiazole derivatives. High temperature exhaustion dyeing was used to apply the dyestuffs to PET fabric as disperse dyestuffs, and the dyed textiles' dye ability, build-up, adsorption isotherms, wash, light, and sublimation fastness were all tested. Furthermore, the effects of medium polarity on the spectrophotometric characteristics of produced dyestuffs with a high polarizability were investigated. They discovered that a considerable bathochromic change in the absorption spectra of all dyestuffs, as seen when changing solvent polarity, which amounts to 82 nm when switching from 1,4-dioxane to DMF, due to a huge difference in the dipole moments of molecules in the ground and excited states. Golshan et al.^30^ studied the optical characteristics and cell imaging capabilities of poly(amidoamine) (PAMAM) dendrimers based on perylene-3,4,9,10-tetracarboxylic diimide (PTCDI). Quantum yield of dendrimers was investigated in different solvents and the highest efficiency was obtained 0.25 in H_2_O solvent. In the near-infrared range, they discovered that manufactured fluorescent dendrimers were absorbent dyes.

Various dendrimers have been synthesized with various core and their fluorescence attributes have been investigated. Song et al.^31^ synthesized three porphyrin-cored dendrimers with non-conjugated coumarins as dendrons. The photophysical properties of dendrimers were investigated in dilute CH_2_Cl_2_ solutions and in thin neat films. The intramolecular energy transfer from the coumarin units to the porphyrin core clearly revealed two factors influencing energy-transfer efficiency. Firstly, a better spectral overlap between the absorption spectrum of porphyrin core and the emission spectrum of the coumarin moiety resulted in high energy-transfer efficiency. Secondly, a long alkyl side-chain improved solubility of dendrimers, whereas prevented the coumarins from self-quenching. Cheng et al.^32^ studied the assembly of coumarin-anchored low generation dendrimers in aqueous solution via hydrophobic interactions. The synthesized material showed significantly improved DNA binding and gene delivery, and minimal toxicity on the transfected cells. Bojinov et al.^33^ designed two novel light-harvesting dendrons with pH sensing properties based on first and second generation PAMAM dendritic scaffolds which surface was labeled with yellow green emitting 1,8-naphthalimide “donor” dyes capable of absorbing light and efficiently transferring the energy to a single Rhodamine 6G “acceptor” dye. They studied the photophysical properties of all synthesized compounds in water/DMF (4:1, v/v) solution and found the core emission intensity of novel systems had enhanced in the pH of 2–6.

The goal of this work is to create fluorescence dendrimers and investigate their characteristics. To do this, poly(amidoamine) (PAMAM) dendrimer (G-0.5) has been prepared by reaction of ethylenediamine (EDA) by methyl acrylate (MA). Then, poly(amidoamine) (PAMAM) dendrimers with EDA core have been synthesized up to 4th generation. Calcozine red 6G and 7-methacryloyloxy-4-methylcoumarin has been conjugated to build a PAMAM dendrimer/7-methacryloyloxy-4-methylcoumarin (G4M32), PAMAM dendrimer/calcozine red 6G (G4R32), and PAMAM dendrimer/7-methacryloyloxy-4-methylcoumarin/calcozine red 6G (G4M16R16). Structure, optical characteristics, and reflectance properties of hybrid dendrimers have been investigated.

## Experimental part

### Synthesis of poly(amidoamine) dendrimer (PAMAM)

PAMAM G4.0 dendrimer was synthesized as described in Supporting Information (S1.2) according to previous works^34,35^.

### Synthesis of 7-methacryloyloxy-4-methylcoumarin (MCMC)

MCMC was synthesized as described in Supporting Information (S1.3) according to previous works^36^.

### Synthesis of hybrid coumarin/PAMAM dendrimer (G4M32)

**Scheme 1:**
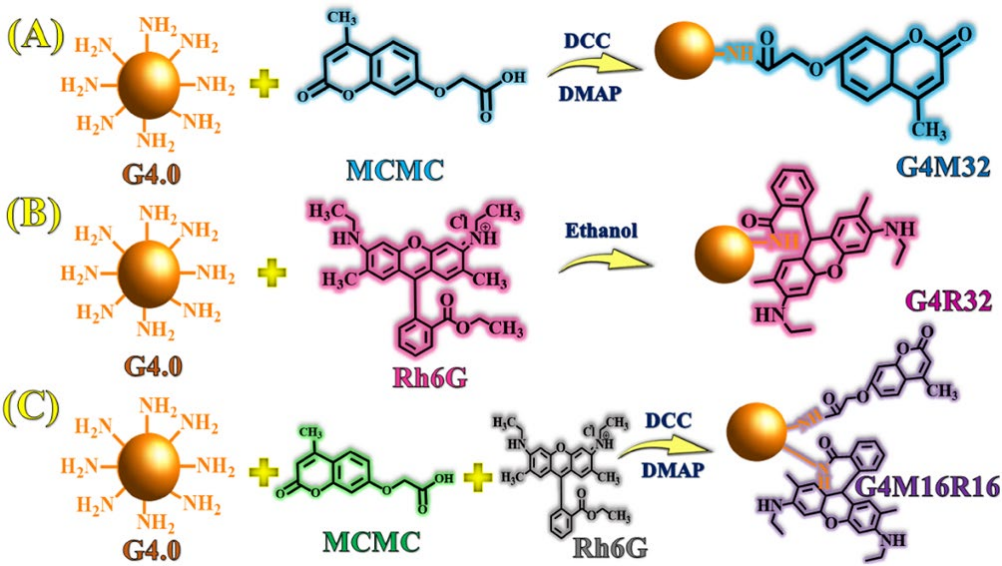
Synthesis mechanism of hybrid dendrimers.

*N*,*N*-Dimethylformamide (20 mL) was used to dissolve MCMC (0.37 g, 1.6 μmol), *N*,*N*′-dicyclohexylcarbodiimide (0.1 g, 0.48 mmol), and 4-(dimethylamino)pyridine (0.02 g, 0.16 mmol). Then, G4.0 (0.7 g, 0.05 mmol) was dissolved in DMSO (5 mL) and dropped into the reaction solution stirring for 168 h at 25 °C under nitrogen (Scheme 1). After reaction completion, the product was washed with 100 mL diethyl ether and dried in a vacuum oven at 60 °C for 48 h. The reaction yield was calculated about 70% gravimetrically.

### Synthesis of hybrid calcozine red 6G/PAMAM dendrimer (G4R32)

G4.0 dendrimer (0.73 g, 0.05 mmol) and calcozine red 6G (0.7 g, 1.6 μmol) were dissolved in ethanol (25 mL). The mixture was heated to 65 °C under nitrogen (Scheme 1). After the reaction was completed, product was rinsed with water (100 mL) and dried in a vacuum oven at 60 °C for 24 h^37^. The reaction yield was calculated ~ 90% gravimetrically.

### Synthesis of hybrid coumarin/calcozine red 6G/PAMAM dendrimer (G4M16R16)

DMF (20 mL) was used to dissolve MCMC (0.19 g, 0.8 mmol), *N*,*N*′-dicyclohexylcarbodiimide (0.1 g, 0.48 mmol), and 4-(dimethylamino)pyridine (0.02 g, 0.16 mmol). After that, G4.0 (0.73 g, 0.05 mmol) was dissolved in DMSO (5 mL) and dropped into the reaction solution, where it was agitated for 168 h at 25 °C under nitrogen atmosphere. After the reaction completion, product (G4M16) was washed with diethyl ether (100 mL) and dried in a vacuum oven at 60 °C for 48 h. Then, calcozine red 6G (0.38 g, 0.8 mmol) and G4M16 (0.91 g, 0.05 mmol) were dissolved in *N*,*N*-dimethylformamide (30 mL). Under nitrogen, the mixture was heated to 120 °C. G4M16R16 was rinsed with water (100 mL) after the reaction was completed (Scheme 1). In a vacuum oven, product was dried at 70 °C for 48 h. The reaction yield was calculated ~ 90% gravimetrically.

### Cell culture and MTT assay

Pasteur Institute provided the SH-SY5Y cell line (Tehran, Iran). The cells were grown and improved in DMEM medium with penicillin (0.1% v/v), FBS (10% v/v), and streptomycin (0.1%v/v) for 24 h in an environment with 95% stickiness and 5% CO_2_. Then, these cells were separated from surface of flask by Trypsin/EDTA and counted. After each brooding period, the medium interior each well is disposed of and put within the hatchery with 10 μl of MTT arrangement (5 mg/ml in PBS) for 4 h. 200 μl of DMSO was included in each well after 4 h and incubated for 24 h at 37 °C. This time provides the opportunity for cells to attach to the plate and multiply. The next day, the medium inside each well was drained and the cells were treated with the desired concentration of dendrimers in a culture medium containing FBS (2%). A group without dendrimer was also considered a control group for comparison. Then, the plates were incubated for 24 h and the viability of the cells after each time was evaluated by MTT assay method. At last, the optical thickness (OD) was examined utilizing the ELIZA Peruser at 570 nm. Equation (1) is used to compute relative cell livability at each concentration and time.

1 $$ {\text{Growth\,inhibition}}\,(\% ) = \left( {1 - \frac{{{\text{OD\,of\,treated\,culture}}}}{{{\text{OD\,of\,control}}}}} \right) \times 100 $$

## Results and discussion

### Photophysical properties of fluorescence dyes

**Figure 1:**
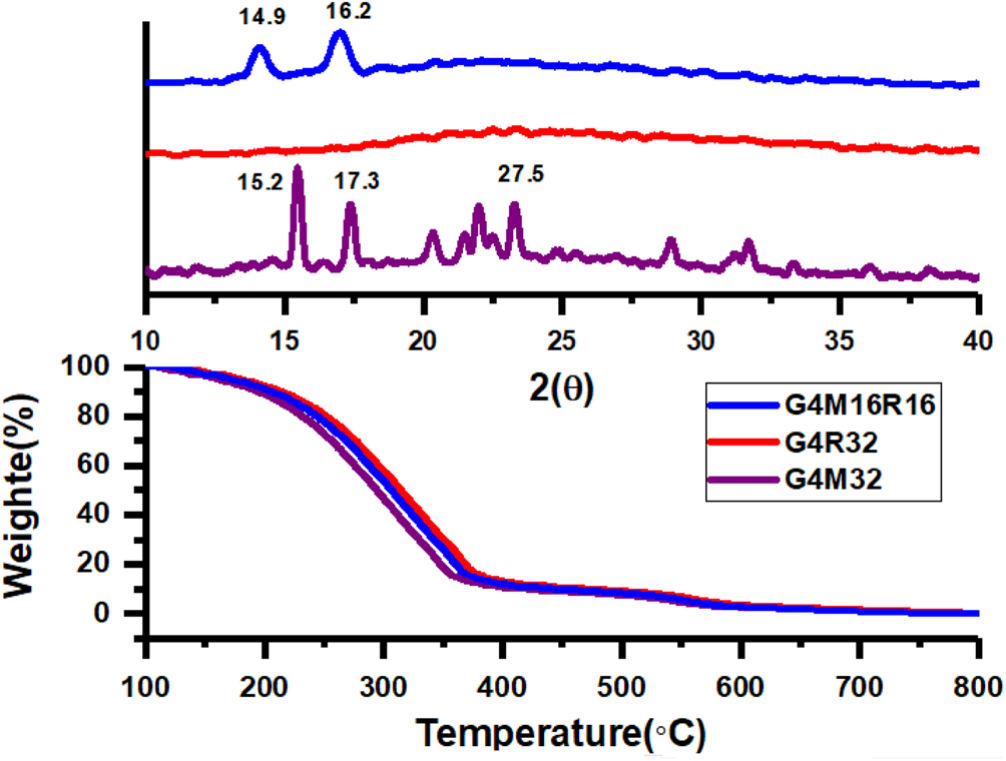
TGA thermograms and XRD patterns of various pigments.

PAMAM dendrimers were synthesized by repeating the Michael addition to methyl acrylate and subsequently amidation with ethylenediamine. As mentioned in Supporting Information, FT-IR, ^1^H NMR, and ^13^C NMR analyses validated synthesis of G4.0 dendrimer (Figures S1–S3). Thermograms of G4M32, G4R32, and G4M16R16 are shown in Fig. 1. Degradation temperature (T_d,max_) and weight loss were determined 350 °C and 88.5% for G4M32, 353 °C and 86.8% for G4R32, and 354 °C and 85.4% for G4M16R16. To determine pigments crystal structure and investigate how different processes affect crystallinity, XRD diffraction patterns were collected (Fig. 1). Peaks at 2θ = 15.2°, 17.3°, and 27.5° are related to coumarin structure in pigments whereas peaks at 2θ = 13.5°, 14.3°, 15.4°, and 16.6° are consistent with Rh6G structure. Thus, defects in crystalline phase exist in all pigments. Crystallinity index of pigments was calculated using Equation (S1)^38^ and obtained 32.1, 29.7, and 30.4% for G4M32, G4R32, and G4M16R16, respectively.

**Figure 2:**
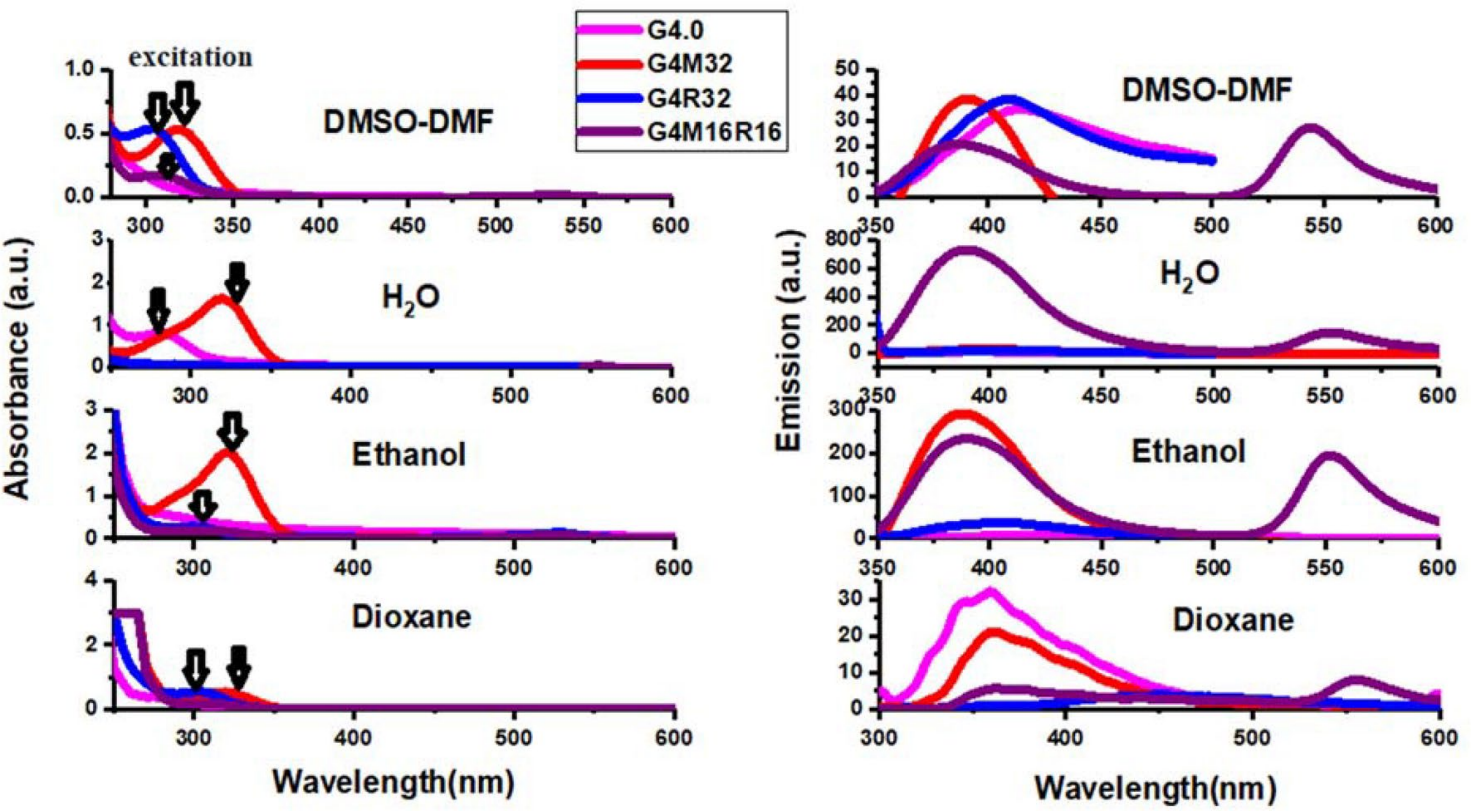
Fluorescence and absorption spectra of hybrid dendrimers.

**Figure 3:**
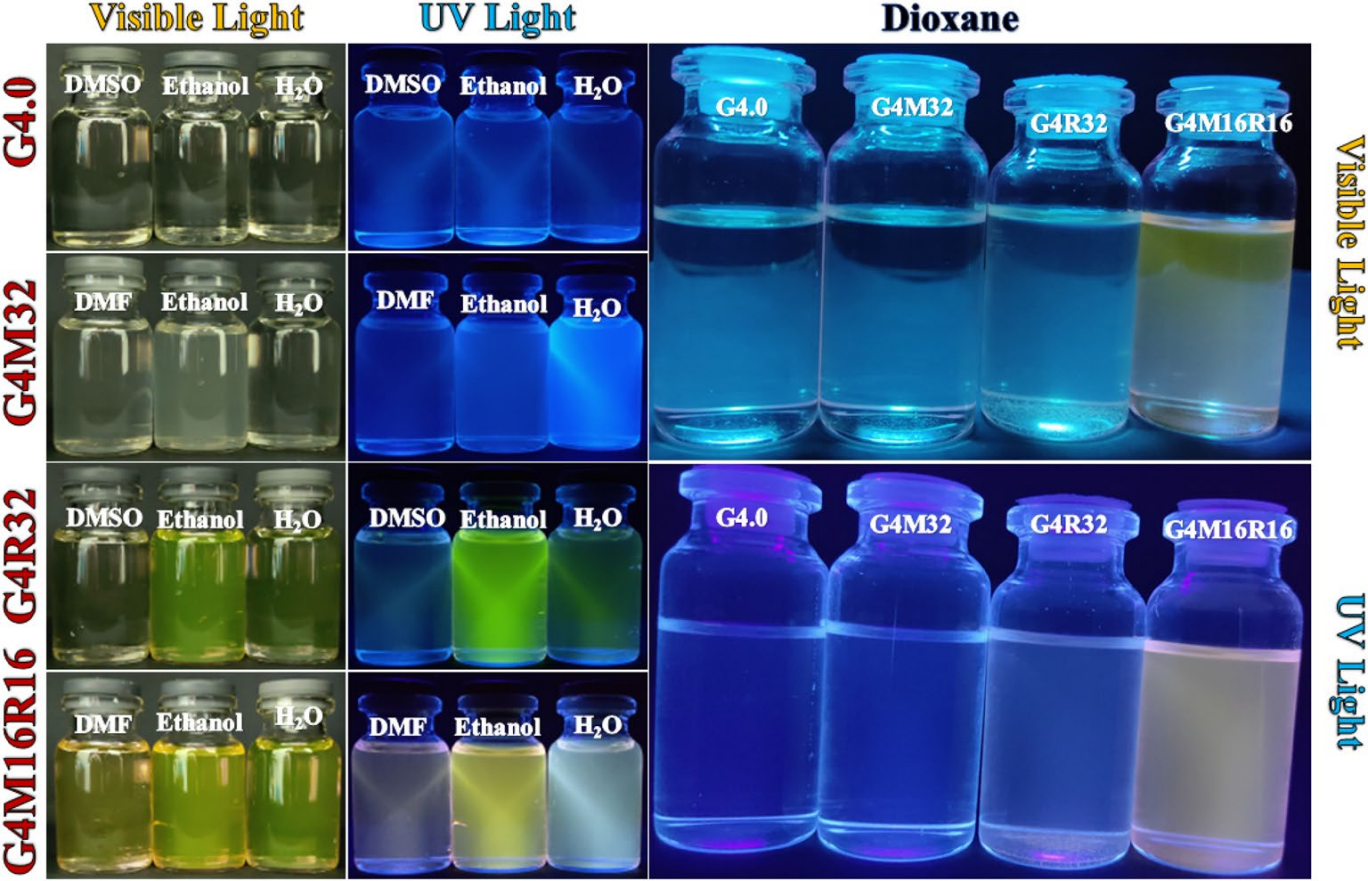
Solution of G4.0, G4M32, G4R32, and G4M16R16 in DMF, Ethanol, DMSO, H_2_O, and 1,4-dioxane.

Some researchers have investigated properties of PAMAM dendrimers using fluorescence probe techniques. Meanwhile, other research groups noticed a weak fluorescence "background" from PAMAM dendrimers. Larson et al.^39^ examined a frail but distinguishable fluorescence emanation from a COO-terminated PAMAM dendrimer by two fluorescence strategies. The observed dendrimer fluorescence appears to be a result of the PAMAM dendrimers' structural peculiarities. When an excitation wavelength in or near the UV area is utilized, emission from the fluorescent moieties inside the dendrimer is seen. An n → π* transition from the amido groups throughout the dendritic structure is the most likely responsible for the faint but discernible fluorescence. The fluorescence emission maxima of all PAMAM samples are at ***λ***_***ex***_ and ***λ***_***em***_ of 380 and 440 nm, respectively, with additional emission band below 320 nm. Excitation-emission matrices (EEMs), a fluorescence method, similarly indicate a rise in relative fluorescence emission with increased generation. For all PAMAM generations, two unique fluorescence lifetimes were discovered. Herein, the excitation and fluorescence spectra of dendrimers and their derivatives are obtained in different solvents (Fig. 2). The highest emission wavelengths for G4.0, G4M32, G4R32, and G4M16R16 were 412, 397, 556, and 553 nm, respectively indicating that the wavelengths of each compound are considerably different. Figure 2 shows the excitation and emission spectra of dendrimers derivatives dissolved in water, ethanol, DMSO, 1,4-dioxane, and DMF at a concentration of 40 μM. In all spectra, various emission wavelengths are observed depending on the solvent (between 396 and 556 nm). In solvents with stronger polarity such as water, a shift to longer wavelengths is observed. The rising polarity of the solvent corresponds to the predicted stability of the excited singlet state's intramolecular charge transfer (ICT) feature in polar liquids. Using Equation (S2), the quantum efficiency of samples was calculated. The largest quantum yield of G4M32 pigment was obtained in water whereas quantum yield in EtOH was quite low. The emission of G4.0, G4M32, G4R32, and G4M16R16 dissolved in DMF, Ethanol, DMSO, 1,4-dioxane, and H_2_O are shown in Fig. 3. Table 1 summarizes spectrum features as well as selected solvent parameters. Peak wavelength and intensity of the spectra are different. This shows that solvent has little effect on the controlling molecular physics of fluorescence. The small changes in solvation of R6G molecules in various solvents might explain deviations^40^.

**Table 1:** Optical properties of hybrid dendrimers.

Sample	*λ*_*ex*_^*a*^	*λ*_*em*_^b^	*λ*_*max*_^c^	Solvent	*Φ*_*s*_^d^
G4.0	285	396	285	H_2_O	0.006
	285	405	285	DMSO	0.003
	285	412	285	Ethanol	0.005
	300	351	300	1,4-dioxane	0.004
G4M32	325	397	325	H_2_O	0.16
	325	397	325	DMF	0.009
	325	397	325	Ethanol	0.008
	310	353	310	Dioxane	0.005
G4R32	530	537	530	H_2_O	0.19
	530	539	530	DMSO	0.08
	530	556	530	Ethanol	0.31
	310	353	310	1,4-dioxane	0.007
G4M16R16	310	553,399	530, 310	H_2_O	0.22
	310	549,399	535,310	DMF	0.13
	310	549,399	535,310	Ethanol	0.28
	310	550, 353	310	1,4-dioxane	0.1

^a^Excitation wavelength, ^b^Maximum emission wavelength, ^c^Maximum absorption wavelength, ^d^Fluorescence quantum yield.

### Cytotoxicity studies

**Figure 4:**
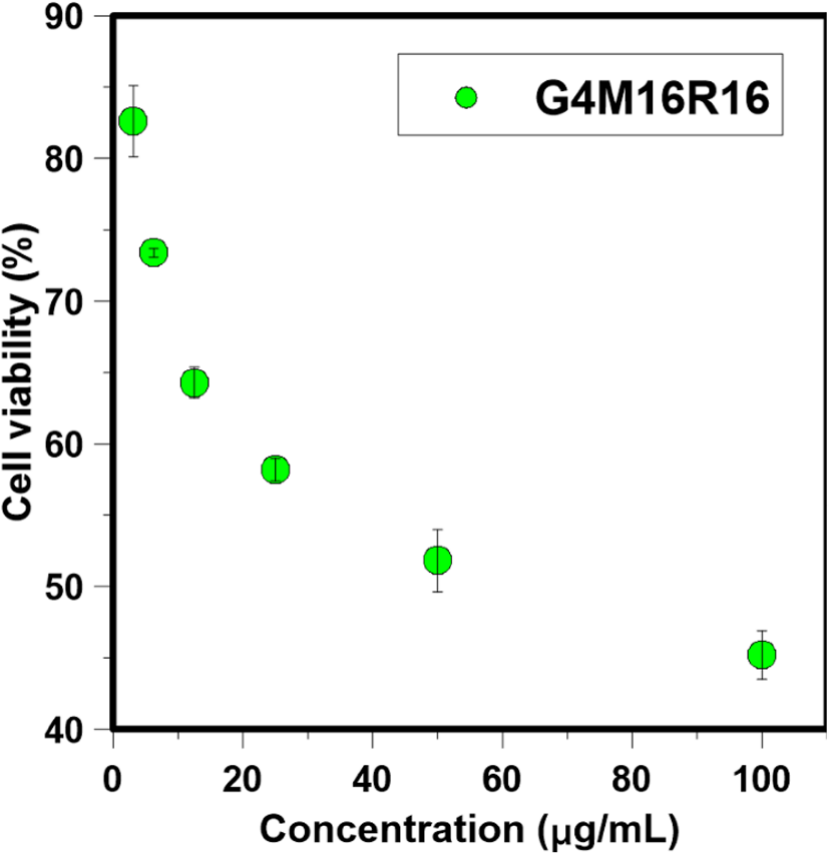
SH-SY5Y cells viability in presence of G4M16R16 pigment after 24 h.

**Figure 5:**
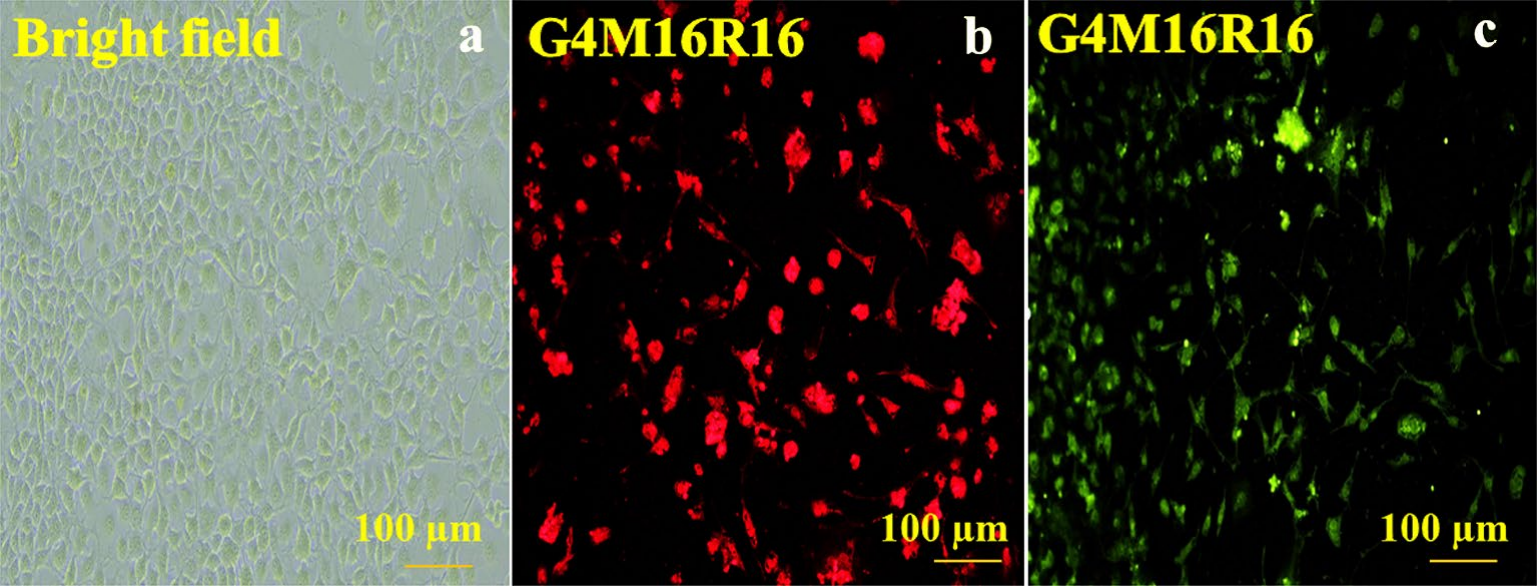
The fluorescence images of hybrid dendrimers-SH-SY5Y at IC50.

Results of MTT assay on SH-SY5Y cells in the presence of G4M16R16 pigment are reported in Fig. 4. At various doses, SH-SY5Y cells the cell viability of these sample was tested after 24 h of incubation. Cell compatibility of fluorescent dendrimer was concentration-dependent and cell viability at concentrations of 3, 6, 12, 25, 50, and 100 μmol L^−1^ was 83.5, 71.4, 66.3, 56.2, 52.8, and 49.2%, respectively. This indicated good cytocompatibility of G4M16R16. IC50 value was calculated using logarithmic regression of cell viability data^41^. Results clearly showed that G4M16R16 had low cytotoxicity effect on SH-SY5Y cell line. IC50 value was obtained 58.4 ± 1.2 μg/mL for G4M16R16 pigment. Then, SH-SY5Y cells were incubated with G4M16R16 solution during 2 h. Figure 5 illustrates that red, and yellow-green fluorescence is clearly located in the cytoplasm of SH-SY5Y cells using a combination of bright field and fluorescence images. Thus, G4M16R16 performed well in live-cell fluorescence imaging.

### NIR reflectance

**Figure 6:**
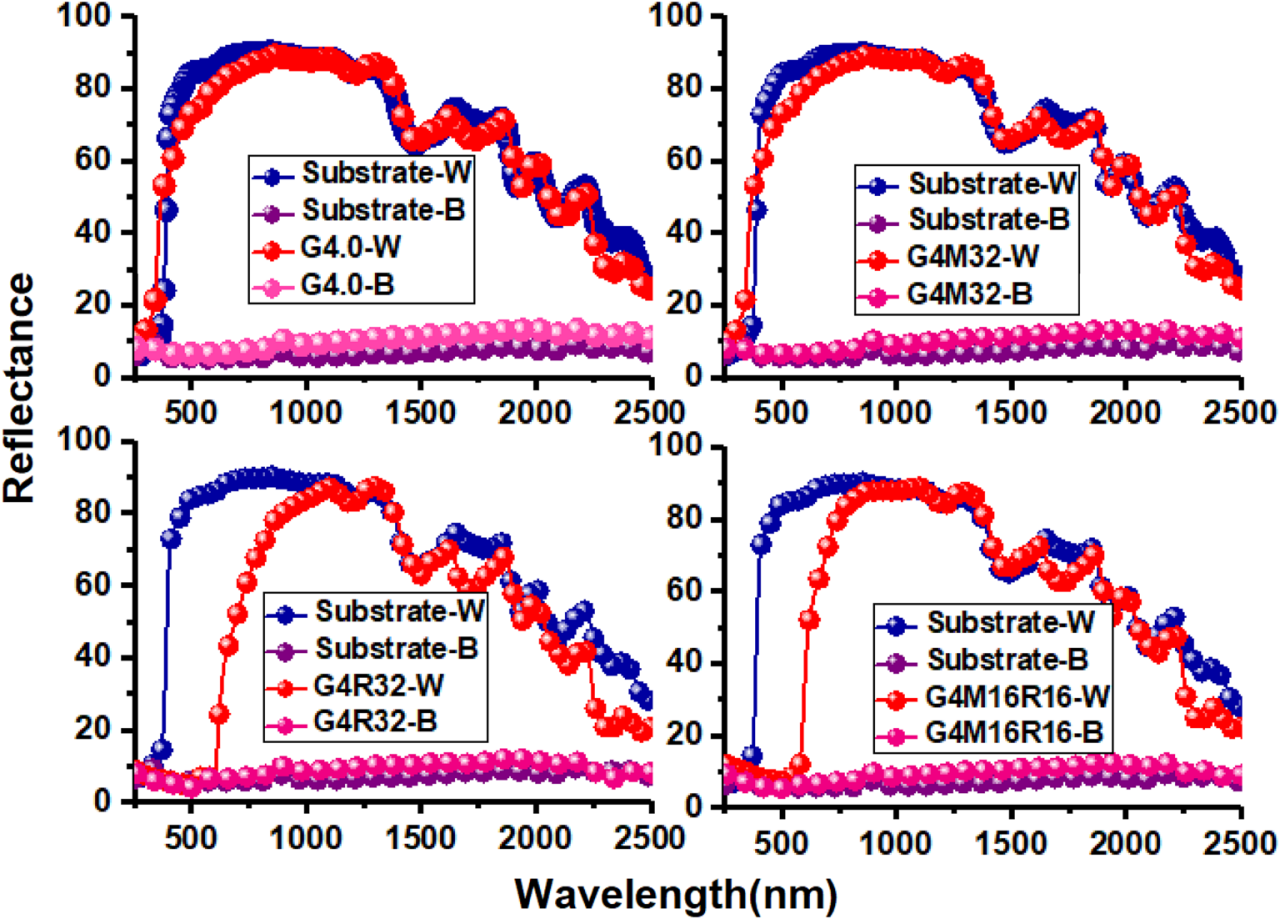
Reflection spectra of hybrid dendrimers.

On two optically different substrates, UV–Vis–NIR reflectance spectra are obtained. The black substrate exhibits substantial absorption at the corresponding wavelengths whereas a white substrate suggests a high reflectivity^42^. The use of sample paints on these optically distinct substrates allows us to study the radiation's transmitted extent qualitatively. In UV–Vis–NIR ranges, all of the samples have varied reflectance patterns, however, the differences in the UV region are minor. Color differences in the pigments correspond to variances in reflectance in the visible region. Figure 6 displays the UV–Vis–NIR spectra of the pigments G4.0, G4M32, G4R32, and G4M16R16 between 250 and 2500 nm. Colors also produced various reflections on the white and black backgrounds. On the black substrate, the reflection rate was less than 20%, but on the white substrate, it was more than 70%. As a result, in the NIR range, G4.0, G4M32, G4R32, and G4M16R16 are categorized as transparent pigments. Integrals of G4.0, G4M32, G4R32, and G4M16R16 pigments were also studied in different regions of UV–Vis–NIR curves, and the results are given in Table 2. In the range of 700–1000 nm, G4.0, G4M32, G4R32, and G4M16R16 exhibited reflections of 98.4%, 98.3%, 83.2%, and 95.7%, respectively, and displayed excellent transparency.

**Table 2:** Reflection of hybrid dendrimers in UV–Vis–NIR areas.

Pigment	700–1000 (nm)	1000–1500 (nm)	1500–2500 (nm)
White substrate	26,819	41,188	55,577
G4.0	26,396	11,512	39,617
G4M32	26,378	41,249	52,796
G4R32	22,317	40,708	47,067
G4M16R16	25,668	41,401	50,654

Pigment	Reflection (%)	Reflection (%)	Reflection (%)
G4.0	98.4%	100%	95.2%
G4M32	98.3%	100%	94.9%
G4R32	83.2%	98.3%	84.6 s%
G4M16R16	95.7%	100%	91.1%

Pigment	700–1000 (nm)	1000–1500 (nm)	1500–2500 (nm)
Black substrate	1981	3297	8223
G4.0	2654	5219	7368
G4M32	2646	5211	12,275
G4R32	2506	4861	10,267
G4M16R16	2532	4906	11,226

Pigment	Reflection (%)	Reflection (%)	Reflection (%)
G4.0	133.9%	158.2%	150.6%
G4M32	133.5%	158.0%	149.2%
G4R32	126.5%	147.4%	124.8%
G4M16R16	127.8%	148.8%	136.5%

## Conclusions

Fluorescent dendrimers have been widely used in various areas, such as optical sensors, drug delivery, gene delivery, cold pigment, and imaging contrast agents. Especially, poly(amidoamine) (PAMAM) dendrimer with facile synthesis, good water solubility, and biocompatibility attracted increasing attention to modifying fluorescence dyes to endow these fluorophores with water solubility and biocompatibility for biologic application. In this study, EDA-cored dendrimer was synthesized iterative sequence Michael addition and amidation reactions up to the fourth generation. Then, PAMAMG4/coumarin (G4M32), PAMAMG4/calcozine red 6G (G4R32), and PAMAMG4/coumarin/calcozine red 6G (G4M16R16) hybrid dendrimers with a perylene core were prepared. Different emission ability was obtained by modifying dendrimer surface with coumarin derivatives and calcozine red 6G. Quantum efficiencies (*φ*_*s*_) of G4.0, G4M32, G4R32, and G4M16R16 at H_2_O, DMF, DMSO, and Ethanol were investigated. Because of the form of ring-opened amide form of calcozine red 6G group and good interaction with ethanol, G4R32 (*ϕs* = 0.31) and G4M16R16 (*ϕs* = 0.28) demonstrated maximum quantum efficiency. Reflectance spectra displayed that G4.0, G4M32, G4R32, and G4M16R16 have a reflectance of 98.4%, 98.3%, 83.2%, and 95.7%in NIR region. G4M32, G4R32, and G4M16R16 according to the results in NIR region are transparent. Cell uptake performance and G4M16R16 fluorescent imaging performance was investigated using neuroblastoma cell lines. Results clearly showed that G4M16R16 has a low cytotoxic effect on SH-SY5Y cell line. Low toxicity of G4M16R16 can be attributed to its hydrophobic components, which prevent direct interaction between G4M16R16 and the cell surface. Red, and yellow-green fluorescence were observed in structure of hybrid pigments due to coumarin derivatives, and calcozine red 6G. Conforming to the results, G4M16R16 pigment has a high ability for live-cell imaging.

## Supplementary Information

Supplementary Information.

## Data Availability

The datasets generated and/or analyzed during the current study are not publicly available at this time as the data form part of an ongoing study. However, the datasets are available from the corresponding author (Mehdi Salami-Kalajahi, m.salami@sut.ac.ir) on reasonable request.
