# Supplementary material for: Synthesis and characterization of fluorescence poly(amidoamine) dendrimer-based pigments
Source: Sci Rep. 2022 Sep 7;12:15180. doi: 10.1038/s41598-022-19712-5 (PMC9452493; doi:10.1038/s41598-022-19712-5)
Supplement: Supplementary file 1 — Supplementary Information. [file 41598_2022_19712_MOESM1_ESM.docx]

**Supporting Information for**

**Synthesis and characterization of fluorescence poly(amidoamine) dendrimer-based pigments**

Marzieh Golshan^1,2^, Behnam Gheitarani^1,2^, Mehdi Salami-Kalajahi^1,2,^*, Mahdi Salami Hosseini^1,2^

^1^ Faculty of Polymer Engineering, Sahand University of Technology, P.O. Box 51335-1996, Tabriz, Iran

^2^ Institute of Polymeric Materials, Sahand University of Technology, P.O. Box 51335-1996, Tabriz, Iran

* Correspondence concerning this article should be addressed to

Mehdi Salami-Kalajahi : Email : m.salami@sut.ac.ir, Tel. /Fax : +98 41 33459097

S1. Experimental part

S1.1. Materials

Ethylenediamine (EDA, Aldrich, 99%), methanol (Merck, 99.9%), methyl acrylate (MA, Aldrich, 99%), nitrocellulose resin, sodium hydroxide (NaOH, Dr. Mojallali, 95%), hydrochloric acid (HCl, Dr. Mojallali, 37%), 4-methylumbelliferone (COUM, Aldrich, 99.5%), potassium carbonate (K_2_CO_3,_ Dr. Mojallali, 99.5%), 4-(dimethylamino)pyridine (DMAP, Aldrich, 99%), N,N*′-*dicyclohexylcarbodiimide (DCC, Merck, 99%), ethyl bromoacetate (Aldrich, 97%), *N*,*N*-dimethylformamide (DMF, DaeJung, 99.5%), dye thinner (Dr. Mojallali, 20000), and calcozine red 6G (Rh6G, Aldrich, 95%) were used as received.

S1.2. Synthesis of poly(amidoamine) dendrimer (PAMAM)

To synthesize PAMAM G4.0 dendrimer, a strategy including repetitive sequence Michael addition and amidation reactions with ethylenediamine as the major core and methyl acrylate was employed (Scheme S1). Half-generations were synthesized by Michael addition reaction of amine end groups and methyl acrylate whereas amidation reaction between half-generations and ethylenediamine was used to synthesize full-generations. These reactions were carried out at ambient temperature under nitrogen and in a dark room. In synthesis of half-generations, 30 *mol*. % excess of MA was used whereas 15–20 molar ratio of [EDA]/[ester groups] was used to synthesize half-generations. After the reaction, solvent was extracted under reduced pressure in a rotary evaporator for 48 h at 50 °C.

S1.3. Synthesis of 7-methacryloyloxy-4-methylcoumarin (MCMC)

Coumarin (39.7 mmol, 7 g) with ethyl bromoacetate (59.6 mmol, 6.5 mL) and anhydrous K_2_CO_3_ (218.3 mmol, 30.0 g) was refluxed for 16 h in acetone (70 mL). The mixture was evaporated to dryness after cooling (Scheme S1). The off-white powder of 4-methyl-7-(methoxy ethyl acetate) coumarin was recrystallized from the residual acetone. 4-methyl-7-(methoxy ethyl acetate) coumarin (26.7 mmol, 7 g) was dissolved in 100 mL ethanol and 42 mL NaOH solution (5%), agitated 24 h at 25 °C, the solvent evaporated, and the residue was diluted in water and acidified with 6 N HCl. The white precipitate was filtered and re-crystallized from ethanol.


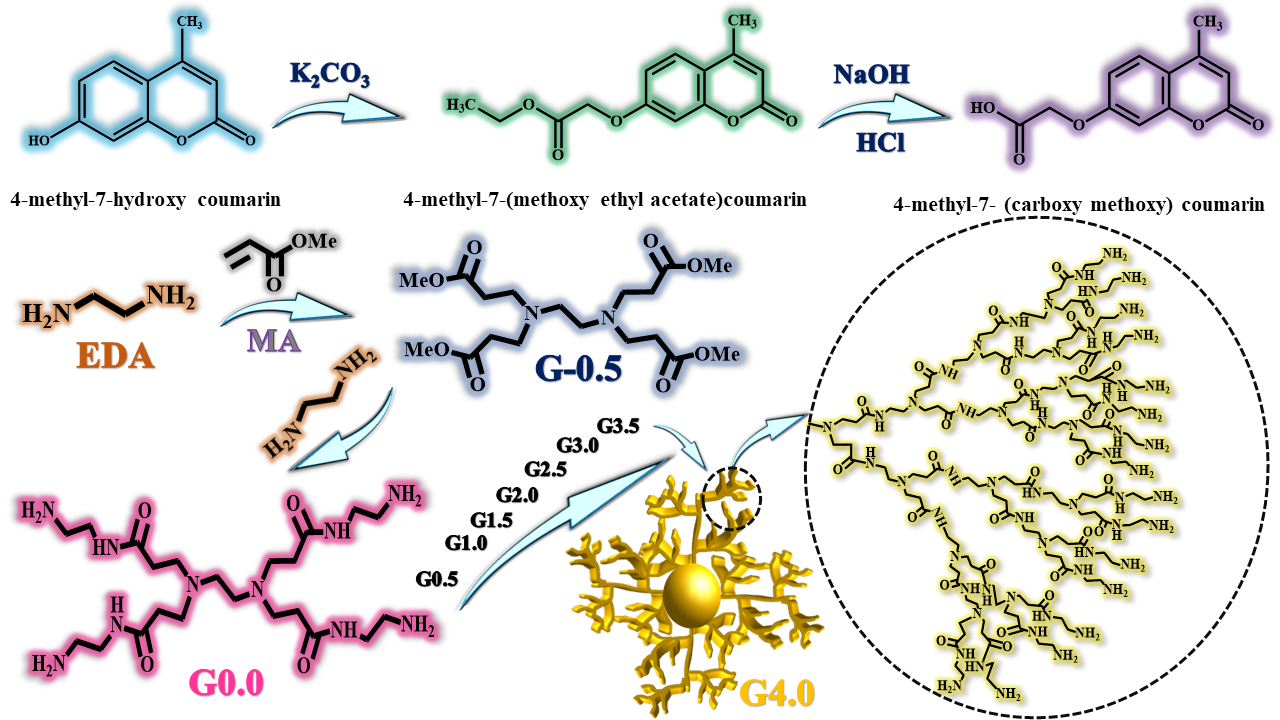


**Scheme S1: Synthesis mechanism of MCMC and PAMAM dendrimer**

S1.4. Structural characterizations

Fourier transform infrared (FT-IR) spectroscopy was recorded on a Bruker Tensor 27 FT-IR spectrophotometer in the wavenumber range of 500 to 4000 cm^−1^ with a resolution of 4 cm^−1^. An average of 24 scans were performed for each sample prepared on a KBr pellet in vacuum desiccators.

The Varian Unity Inova model (500 MHz) proton and carbon nuclear magnetic resonance (^1^H NMR) were used to verify accuracy of synthesis in each step using deuterated DMSO (DMSO-*d*_6_), and D_2_O as solvents.

Ultraviolet visible (UV–visible) absorption spectra from liquid samples were recorded by a Hanon instrument.

Fluorescence spectrophotometer (FS, PerkinElmer LS-45) was used to determine the emission rate of pigments.

X-ray diffraction (XRD) was used to investigate the structural properties of produced films. XRD spectra were collected on an X-ray diffraction instrument (Siemens D5000) with a Cu target (λ = 0.1540 nm) at room temperature. The system consists of a rotating anode generator which operated at 35 kV and 20 mA. The samples were scanned from 2θ = 10 to 40° at the step scan mode; the diffraction pattern was recorded using a scintillation counter detector.

UV–Vis–NIR reflectance spectra were obtained over the wavelength range of 250–2500 nm using a PerkinElmer lambda 1050.

Thermal gravimetric analyses (TGA) were carried out by means of a PL thermo-gravimetric analyzer (Polymer Laboratories, TGA 1000, UK). The pigments (about 10 mg) were heated from ambient temperature to 700 °C at a heating rate of 10 °C/min and nitrogen as the purging gas was used at a flow rate of 50 mL/min.

S1.5. Dye preparation

Nitrocellulose resin (64 *wt*. %), pigments (16 *wt*. %), and solvent (20 *wt*. %, dye thinner) were mixed and ball-milled for 5 h. Then, resultant paints were applied to Leneta checkerboard charts using a film applicator with a thickness of 120 µm. This helped to prevent reflection of the substrate and only reflection of paint was detected. Evaluation of pigments reflectance on a highly reflective substrate (white substrate) and highly absorbent substrate (black substrate) can qualitatively show the amount of reflection, transfer, and absorption of pigment.

S2. Results

S2.1. Spectral characterization

**G0.0:**

**FT-IR main absorption peaks of G0.0 (υ/cm^-1^, Figure S1):** 1640 (C=O), 3200 (NH_2_), 3400 (NH).

**^1^H NMR of G0.0 (500 MHz, D_2_O, δ/ppm, Figure S2):** 2.5 (4***H***, s, a), 2.7 (8***H***, t, b), 2.5 (8***H***, t, c), 7.9 (4***H***, bt, d), 3.3 (8***H***, bq, e), 2.2 (8***H***, p, f), 2.4 (8***H***, t, g).

**^13^C NMR of G0.0 (500 MHz, D_2_O, δ/ppm):** 41.0 (***C***H_2_, a), 49.2 (***C***H_2_, b), 35.6 (***C***H_2_, c), 175.6 (***C***O, d), 40.6 (***C***H_2_, f), 39.8 (***C***H_2,_ g).

**G1.0:**

**FT-IR main absorption peaks of G1.0 (υ/cm^-1^, Figure S1):** 1640 (C=O), 3150 (NH_2_), 3420 (NH).

**^1^H NMR of G1.0 (500 MHz, D_2_O, δ/ppm, Figure S2):** 2.5–2.6 (52***H***, m, a, b, c, g, h), 7.9 (12***H***, bt, d, i), 3.1–3.3 (24***H***, bq, e, j), 2.6 (8***H***, t, f), 2.2 (16***H***, bp, k), 2.4 (16***H***, t, l).

**^13^C NMR of G1.0 (500 MHz, D_2_O, δ/ppm):** 41.0 (***C***H_2_, a, f, g), 49.2 (***C***H_2_, b, h), 35.6 (***C***H_2_, c, i), 175.6 (***C***O, d, j), 40.6 (***C***H_2_, f, l), 39.8 (***C***H_2,_ g, m).

**G2.0:**

**FT-IR main absorption peaks of G2.0 (υ/cm^-1^, Figure S1):** 3275 (NH_2_).

**^1^H NMR of G2.0 (500 MHz, D_2_O, δ/ppm, Figure S2):** 2.6–2.8 (116***H***, bm, a, b, c, g, h, l, m), 7.8 (28***H***, bm, d, i, n), 3.1–3.4 (56***H***, bm, e, j, o), 2.9–2.9 (24***H***, bm, f, k), 2.2 (32***H***, bp, p), 2.4 (32***H***, bt, q).

**^13^C NMR of G2.0 (500 MHz, D_2_O, δ/ppm):** 41.0 (***C***H_2_, a, f, g), 49.2 (***C***H_2_, b, h, n), 35.6 (***C***H_2_, c, i, o), 175.6 (***C***O, d, j, p), 40.6 (***C***H_2_, f, l, r), 39.8 (***C***H_2,_ g, m, s).

**G3.0:**

**FT-IR main absorption peaks of G3.0 (υ/cm^-1^, Figure S1):** 1650 (C=O), 3265 (NH_2_).

**^1^H NMR of G3.0 (500 MHz, D_2_O, δ/ppm, Figure S2):** 2.6–2.8 (244***H***, bm, a, b, c, g, h, l, m, q, r), 7.85 (60***H***, bm, d, i, n, s), 3.1–3.4 (120***H***, bm, e, j, o, t), 2.5–2.7 (56***H***, bm, f, k, p), 2.2 (64***H***, bp, u), 2.4 (64***H***, bt, v).

**^13^C NMR of G3.0 (500 MHz, D_2_O, δ/ppm):** 41.0 (***C***H_2_, a, f, g), 49.2 (***C***H_2_, b, h, n,t), 35.6 (***C***H_2_, c, i, o, u), 175.6 (***C***O, d, j, p,v), 40.6 (***C***H_2_, f, l, r, x), 39.8 (***C***H_2,_ g, m, s,y).

**G4.0:**

**FT-IR main absorption peaks of G4.0 (υ/cm^-1^, Figure S1):** 1650 (C=O), 3265 (NH_2_).

**^1^H NMR of G4.0 (500 MHz, D_2_O, δ/ppm, Figure S2):** 2.7–2.9 (500***H***, bm, a, b, c, g, h, l, m, q, r, v, w), 7.9 (124***H***, bm, d, i, n, s, x), 3.0– 3.3 (248***H***, bm, e, j, o, t, y), 2.4–2.6 (120***H***, bm, f, k, p, u), 2.1–2.2 (128***H***, bp, z), 2.4–2.4 (128***H***, bt, α).

**^13^C NMR of G4.0 (500 MHz, D_2_O, δ/ppm, Figure S3):** 41.0 (***C***H_2_, a, f, g), 49.2 (***C***H_2_, b, h, n, t, z), 35.6 (***C***H_2_, c, i, o, u, a^/^), 175.6 (***C***O, d, j, p, v, b^/^), 40.6 (***C***H_2_, f, l, r, x, d^/^), 39.8 (***C***H_2,_ g, m, s, y, e^/^).

**G4M32:**


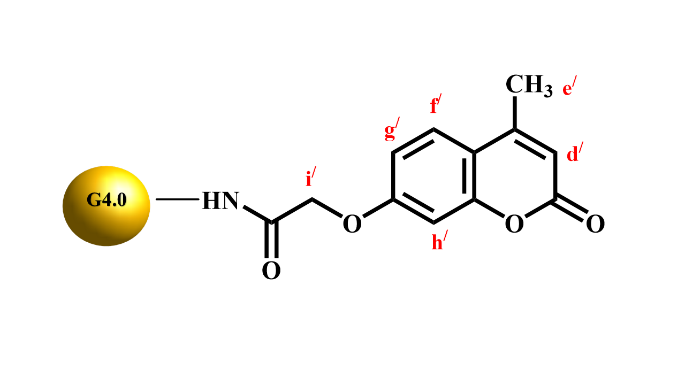


**FT-IR of G4M32 (KBr, cm^-1^, Figure S1):** 1278 (C=O), 3192 (υNH_2_), 3400 (υNH), (2987, 2916 assym. str. CH_3_, CH_2_), (1755 C=O), (1708 C=O), (1610, 1566, 1510 str. C=C), (1427, 1390 CH_3_, CH_2_ bend).

**^1^H NMR of G4M32** **(500 MHz, DMSO, δ/ppm, Figure S2):** 2.4 (bt, 128***H***, b^/^), 2.39 (s, 3***H***, e^/^), 4.7 (s, 2***H***, i^/^), 6.0 (s, 1***H***, d^/^), 6.2 (s, 1***H***, g^/^), 6.9 (d, 1***H***, h^/^), 7.6 (d, 1***H***, f^/^).

**^13^C NMR of G4M32 (500 MHz, DMSO, δ/ppm, Figure S3):** 112.5 (d^/^), 19.4 (e^/^), 125.8 (**f**^/^), 110 (g^/^), 104 (i^/^).

**G4R32:**


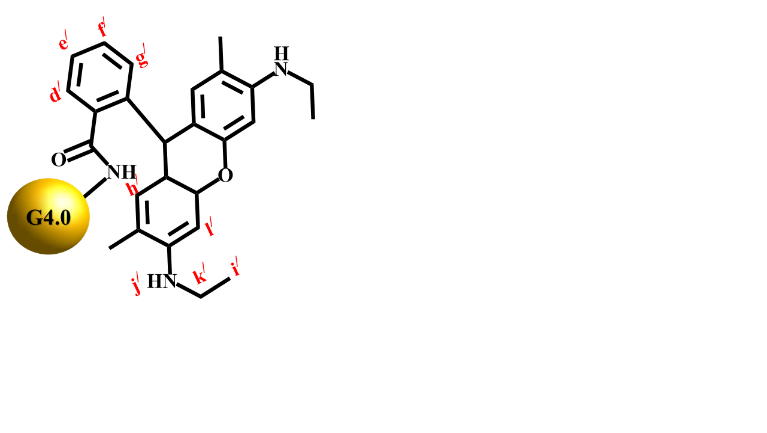


**FT-IR of G4R32 (KBr, cm^-1^, Figure S1):** 3250 (υNH_2_), 2860-2960 (υCH).

**^1^H NMR of G4R32** **(500 MHz, DMSO, δ/ppm, Figure S2):** 7.8 (m, 2***H***, d^/^), 7.4 (m, 8***H***, e^/^, f^/^), 7.1 (s, 2***H***, g^/^), 6.4 (m, 4***H***, h^/^), 6.4 (m, 4***H***, l^/^), 6.2 (m, 4***H***, j^/^).

**^13^C NMR of G4R32 (500 MHz, DMSO, δ/ppm, Figure S3):** 16 (i^/^), 38.7 (k^/^), 103.0 (**l**^/^), 124.9 (g^/^), 132.6 (f^/^), 129.2 (d^/^).

**G4M16R16:**


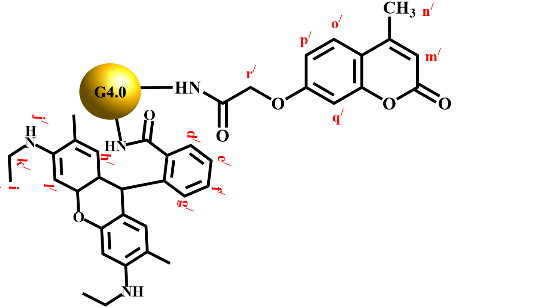


**FT-IR of G4M16R16 (KBr, cm^-1^, Figure S1):** 3192 (υNH_2_), 3400 (υNH), (2987, 2916 assym. str. CH_3_, CH_2_), (1755 C=O), (1708 C=O), (1610, 1566, 1510 str. C=C), (1427, 1390 CH_3_, CH_2_ bend), 3250 (υNH_2_), 2860-2960 (υCH).

**^1^H NMR of G4M16R16** **(500 MHz, DMSO, δ/ppm, Figure S2):** 7.8 (m, 2***H***, d^/^), 7.4 (m, 8***H***, e^/^, f^/^), 7.1 (s, 2***H***, g^/^), 6.4 (m, 4***H***, h^/^), 6.4 (m, 4***H***, l^/^), 6.2 (m, 4***H***, j^/^), 2.3 (s, 3H, n^/^), 4.7 (s, 2***H***, r^/^), 6.0 (s, 1***H***, m^/^), 6.2 (s, 1***H***, p^/^), 6.9 (d, 1***H***, q^/^), 7.6 (d, 1***H***, o^/^).

**^13^C NMR of G4M16R16 (500 MHz, DMSO, δ/ppm, Figure S3):** 112.5 (m^/^), 19.4 (n^/^), 125.8 (**o**^/^), 110 (p^/^), 104 (r^/^), 16 (i^/^), 38.7 (k^/^), 103.0 (**l**^/^), 124.9 (g^/^), 132.6 (f^/^), 129.2 (d^/^).


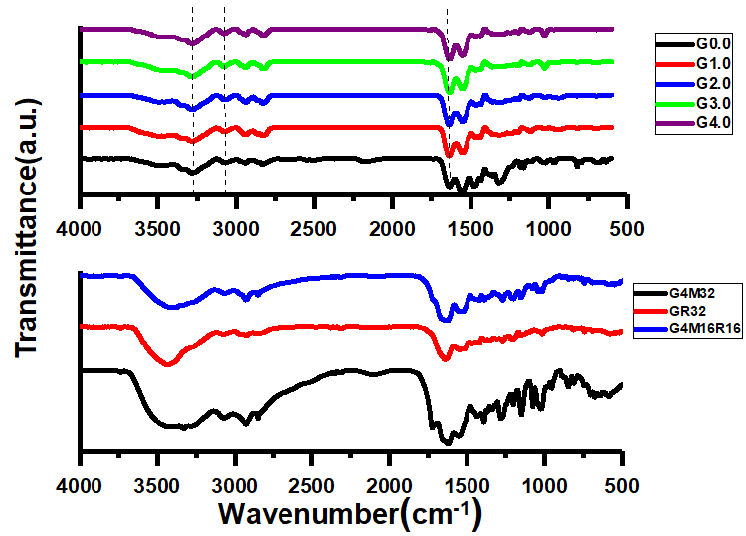


**Figure S1: FTIR spectra of various dendrimers**


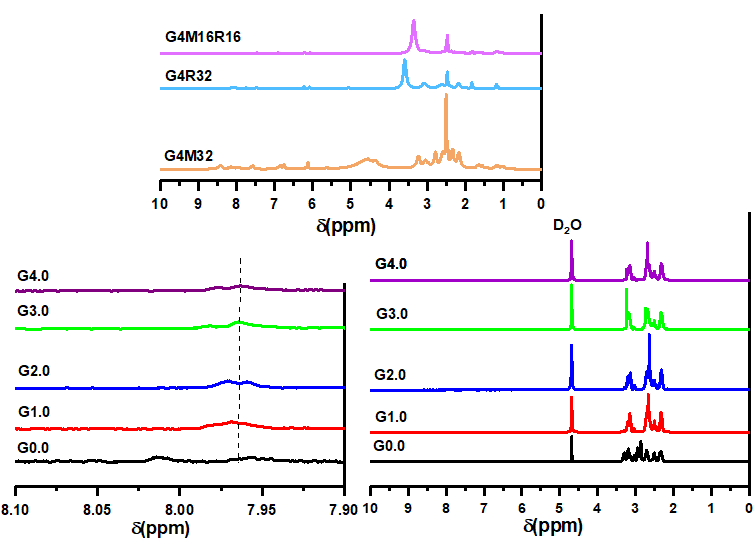


**Figure S2: ^1^H NMR spectra of hybrid dendrimers**


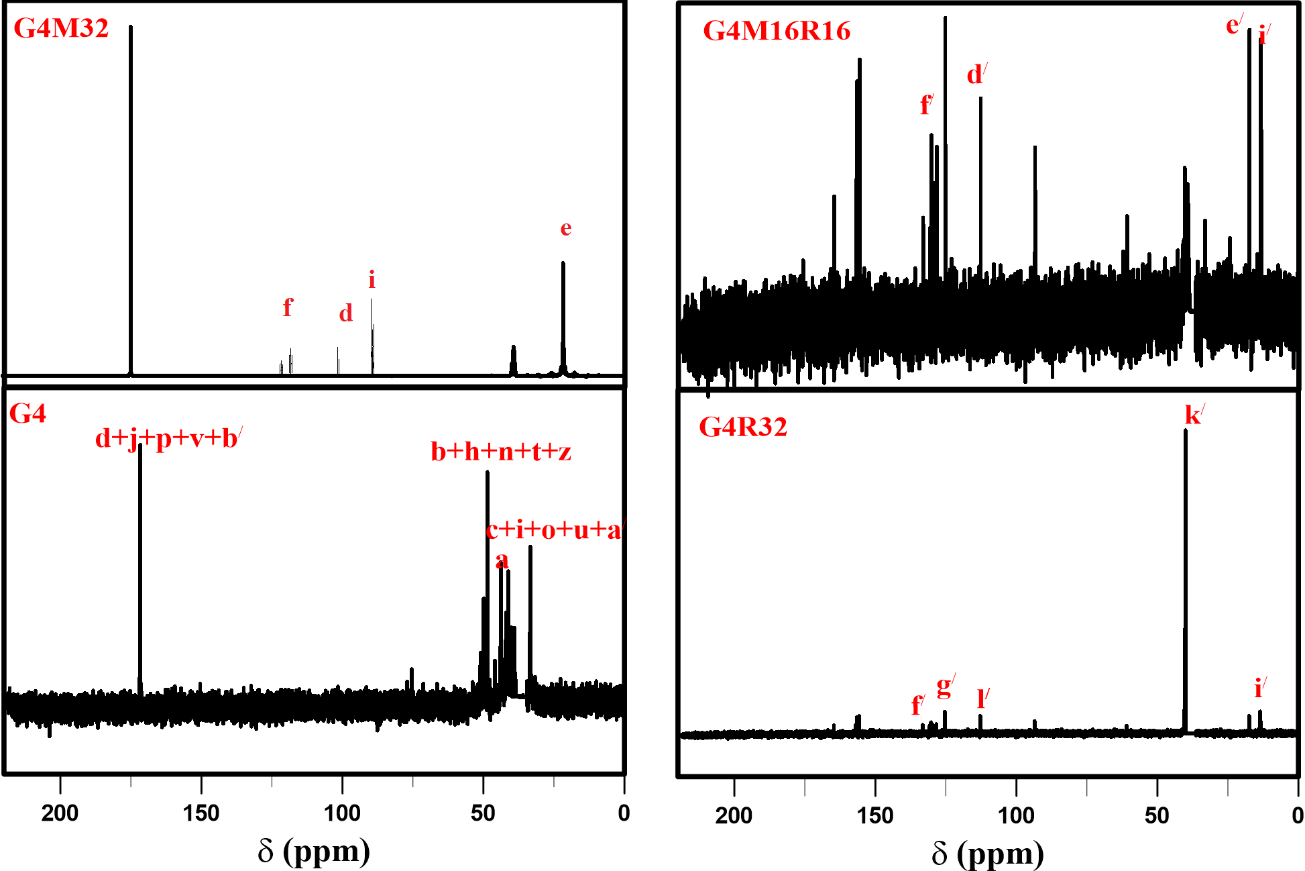


**Figure S3: ^13^C NMR spectra of hybrid dendrimers**

S2.2. Equations

$\text{Crystallinity (\%) = }\frac{\text{Total area of crystalline peaks}}{\text{Total area of all peaks}}\text{ }\times100$ (S1)

$\Phi_{s}=\Phi_{r}\frac{m_{s}}{m_{r}}\left(\frac{\eta_{s}}{\eta_{r}}\right)^{2}$ (S2)

In this equation, *Φ_r_* is the quantum yield of the standard pigment (An ethanol solution of rhodamine 6G was used as a standard for measuring the luminescence quantum yield (the quantum yield in an ethanol solution is 95%)), *m_s_* is the slope of the linear fit for the integrated fluorescence intensity of the fluorescent pigment as a function of absorbance, and *η_s_* and *η_r_* are the refractive indices of the fluorescent pigment and the standard solutions, respectively.
